# Targeted protein degradation: mechanisms, strategies and application

**DOI:** 10.1038/s41392-022-00966-4

**Published:** 2022-04-04

**Authors:** Lin Zhao, Jia Zhao, Kunhong Zhong, Aiping Tong, Da Jia

**Affiliations:** 1 Key Laboratory of Birth Defects and Related Diseases of Women and Children, Department of Paediatrics, West China Second University Hospital, State Key Laboratory of Biotherapy and Collaborative Innovation Center of Biotherapy, Sichuan University, 610041 Chengdu, China; 2 State Key Laboratory of Biotherapy and Cancer Center, West China Hospital, West China Medical School, Sichuan University, 610041 Chengdu, China

**Keywords:** Chemical biology, Molecular medicine

## Abstract

Traditional drug discovery mainly focuses on direct regulation of protein activity. The development and application of protein activity modulators, particularly inhibitors, has been the mainstream in drug development. In recent years, PROteolysis TArgeting Chimeras (PROTAC) technology has emerged as one of the most promising approaches to remove specific disease-associated proteins by exploiting cells’ own destruction machinery. In addition to PROTAC, many different targeted protein degradation (TPD) strategies including, but not limited to, molecular glue, Lysosome-Targeting Chimaera (LYTAC), and Antibody-based PROTAC (AbTAC), are emerging. These technologies have not only greatly expanded the scope of TPD, but also provided fresh insights into drug discovery. Here, we summarize recent advances of major TPD technologies, discuss their potential applications, and hope to provide a prime for both biologists and chemists who are interested in this vibrant field.

## Protein degradation pathways: proteasomal and lysosomal pathways

Protein homeostasis, also known as proteostasis, refers to a highly complex and interconnected process used by cells to maintain concentration, conformation, and subcellular localization of proteins.^1^ It comprises a large set of pathways that control protein synthesis, folding, protein transport, and disposal.^2^ In eukaryotic cells, damaged proteins or organelles can be cleared by proteasomes or lysosomes.^3,4^ The two pathways are independent but inter-connected with each other.^5^ In general, proteasomes eliminate short-lived proteins and soluble misfolded proteins by the ubiquitin–proteasome system (UPS).^6,7^ In contrast, lysosomes are responsible for degradation of long-lived proteins, insoluble protein aggregates, even entire organelles, macromolecular compounds, and intracellular parasites (e.g. certain bacteria) via endocytosis, phagocytosis, or autophagy pathways.^8–13^

**Fig. 1:**
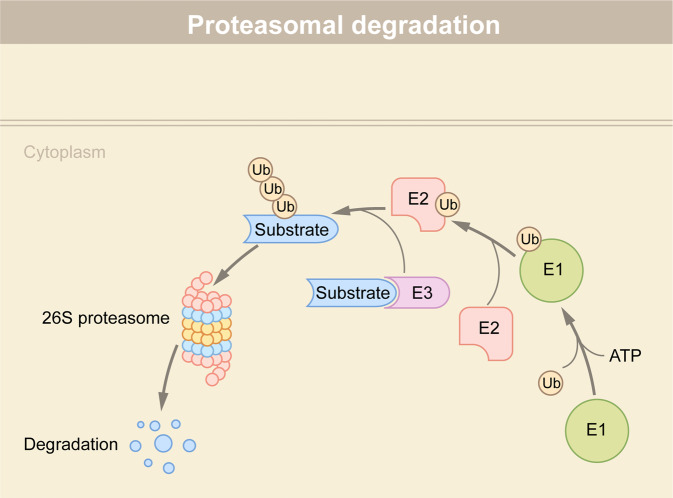
Protein degradation via the ubiquitin-proteasome system (UPS). Proteins undergo ubiquitin-dependent degradation by a suite of three enzymes. E1 interacts with E2, and transfers the ubiquitin molecule to E2. E2 interacts with E3-binding substrate and transfers the ubiquitin molecule to the substrate. Repetition of these processes results in polyubiquitination of the substrate, which is subsequently degraded by the 26S proteasome

Proteasomes are part of the UPS responsible for degradation of proteins that are damaged, unfolded, and useless.^14^ In addition to proteasomes, the UPS also compromises various ubiquitin ligases and de-ubiquitinating enzymes (DUBs).^15^ The 76-residue ubiquitin protein is attached to proteins via a lysine isopeptide bond as a post-translational modification (PTM) through sequential reaction involving three enzymes: a Ub activating enzyme(E1), a Ub conjugating enzyme(E2), and a Ub ligase (E3)^16,17^ (Fig. 1). E1 binds to the ubiquitin molecule in an ATP-dependent manner and then transfers it to E2 via an interaction with E2. Next, E3 catalyzes the transferring of the ubiquitin molecule from E2 to substrates.^18^ The repeated action of these three enzymes lead to the polyubiquitination of the substrate. There are eight different polyubiquitin chains (seven lysine residues: K6, K11, K27, k29, k33, K48, k63 and one methionine residue) depending on the residue number of the ubiquitin molecule that is conjugated.^19^ Among them, K48 and K63 linkages are the most abundant and account for ~80% of total linkages in mammalian cells. Proteins marked with K48-linked ubiquitin chains are often targeted to proteasome for degradation; in contrast, K63-linked chains do not function in proteasomal degradation, but play a pivotal role in regulating lysosome functions and inflammatory response.

**Fig. 2:**
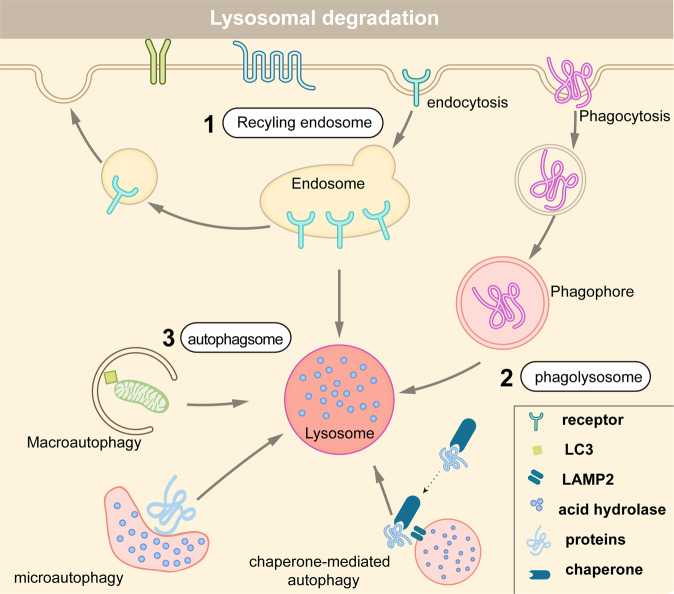
Protein degradation via three distinct lysosome pathways. (1) Cell surface proteins arrive at endosome after endocytosis. They could be degraded by lysosome, or transported to the plasma membrane or other cellular organelles for recycling. (2) In the phagocytic pathway, cells engulf large extracellular particles, such as invading pathogens and dead cells, and then degrade them by lysosome. (3) Misfolded or aggregated proteins, damaged organelles, and intracellular pathogens, are removed by the autophagy–lysosome pathway. There are three different forms of autophagy: macroautophagy, microautophagy, and chaperone-mediated autophagy

Lysosomes are the primary degradative compartments of the cells, and receive their degradation substances via endocytosis, phagocytosis, or autophagy^20^ (Fig. 2). Following endocytosis, some cell surface proteins are recycled to the plasma membrane or other organelles, whereas others are marked with K63-linked ubiquitin chains and sorted into the endosomal sorting complex required for transport (ESCRT) complex degradation pathway.^21–24^ Phagocytosis is a specific form of endocytosis by which cells engulf microbial pathogens or other large particles.^25^ Finally, autophagy is an evolutionarily conserved process that cells use to remove unnecessary or dysfunctional intracellular organelles and proteins through a lysosome-dependent manner. Targeted organelles and proteins are wrapped into a double membrane-bound vesicle, known as autophagosome.^26^ The autophagosome then fuses with lysosomes to break down the contents.^27,28^

**Fig. 3:**
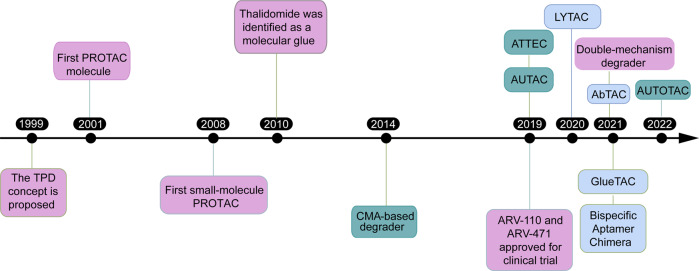
Representative events in the TPD development. Purple: technologies related with UPS-based technologies; light blue: technologies related with the endosome-lysosome pathway; dark blue: technologies related with the autophagy-lysosome pathway

Targeted protein degradation (TPD), via the proteasomal and lysosomal pathways, represent a novel tool to explore cellular pathways and a promising therapeutic approach.^29^ The concept of TPD was first proposed in 1999 (Fig. 3). Most TPD strategies, such as PROTACs,^30,31^ molecular glues,^32^ degradation tags (dTAGs),^33^ trim away,^34^ and specific and non-genetic inhibitors of apoptosis protein-dependent protein erosive agents (SNIPERs),^35^ rely on the UPS and mainly target intracellular proteins. Lysosome-dependent TPD strategies could degrade membrane proteins, extracellular proteins, and protein aggregates, thus greatly expanding the range of substrates. In this review, we first provide a simple introduction of protein degradation mechanisms. We will then summarize recent advances in developing various TPD technologies, and highlight their potential applications in disease treatment. Interested readers are encouraged to read other excellent reviews that cover other aspects of the field.^36–41^

## Targeted protein degradation via proteasome

In the canonical ubiquitination pathway, ubiquitin is conjugated to target proteins by an E1-E2-E3 enzymatic cascade (Fig. 1). As the E3 ligase is responsible for recognizing substrates and its family number greatly exceeds E1 and E2, the UPS-based TPD strategies utilize E3 ligases as targeting proteins for degradation.^42^ PROTAC and molecular glue are two major technologies that rely on the UPS for the degradation of protein of interest (POI), and will be the focus of our discussion (Table 1). Additionally, many PROTAC-based technologies, including selective androgen receptor degrader (SARD)^43,44^, Hydrophobic tagging (HyT),^45–47^ TF-PROTAC,^48^ dual-PROTAC,^49^ and selective estrogen receptor degrader (SERD),^50–54^ have recently emerged (Table 1).

**Table 1:** Different TPD technologies

Degradation pathways	Degradation system	Technologies	Refs.
Targeted protein degradation via proteasome	ubiquitin–proteasome system (UPS)	PROTAC	^31,58^
		Molecular glue	^69^
		Double-mechanism degrader	^71^
		PROTAC-based technologies: SARD, HyT, TF-PROTAC, dual-PROTAC, SERD	^43–54^
Targeted protein degradation via lysosome	endosome-lysosome system	LYTAC	^81,82^
		Bispecific Aptamer Chimera	^79^
		AbTAC	^86^
		GlueTAC	^88^
	autophagy-lysosome system	AUTAC	^93,94^
		ATTEC	^96,97^
		AUTOTAC	^80^
		CMA-based degrader	^105^

### PROTAC

**Fig. 4:**
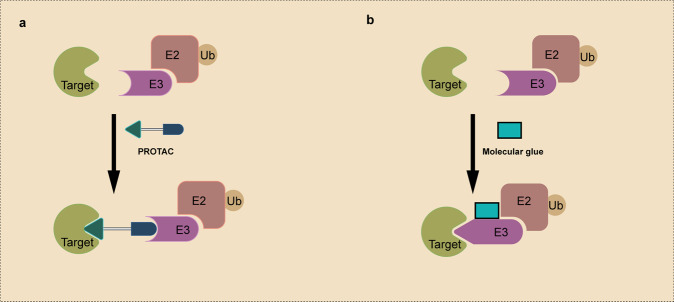
Schematic representation of PROTAC and Molecular Glue. **a** A PROTAC molecule consists of an E3 ligase-targeting ligand, a linker, and a POI-binding ligand. It simultaneously binds to the POI and the E3 ubiquitin ligase, and induces the polyubiquitination and degradation of the POI. **b** Molecular glue induces the interaction between a POI and an E3 ubiquitin ligase via binding to the E3 ubiquitin ligase, as illustrated, or the POI. Relative to PROTAC molecules, molecular glues do not have a linker and have a lower MW

A PROTAC molecule comprises an E3-recruiting ligand, a POI-targeting warhead, and a flexible linker linking the two ligands (Fig. 4a). The addition of PROTAC promotes the formation of the POI-PROTAC-E3 ternary complex, induces ubiquitination of the POI and subsequent degradation via the UPS.^37,55,56^ Crews and Deshaies groups developed the first PROTAC molecule in 2001.^31^ The protein-targeting chimeric molecule 1 (Protac-1) was synthesized to recruit target protein methionine aminopeptidase-2 (MetAP-2) to the Skp1-Cullin-F-box (SCF) ubiquitin ligase complex, and subsequently degraded^31^(Fig. 3). The Protac-1 contains two domains: one domain consists of a phosphopeptide derived from IkBα (IPP) and binds to SCF, and the other domain, composed of ovalicin, interacts with MetAP-2.^31^ Subsequently, the same group demonstrated that a chimeric molecule consisting of the IκB phosphopeptide and small-molecules could be used to degrade the estrogen receptor (ER) and androgen receptor (AR), which promote the growth of breast and prostate cancers, respectively.^57^

In 2008, Crews’s group reported the first example of small molecule-based PROTAC^58^ (Fig. 3). This PROTAC, consisting of nonsteroidal androgen receptor ligand (SARM), a MDM2 ligand targeting ubiquitin ligase murine double minute 2 (MDM2), and a PEG-based linker, was used to degrade androgen receptor (AR).^58^ In comparison with peptide-based PROTACs, small molecule PROTACs are more readily taken up by cells and more likely to be developed into drugs.^59^ In addition to MDM2, multiple other E3 ligases have been harnessed in the PROTAC technology, including cereblon (CRBN),^60^ Von-Hippel-Lindau (VHL),^61^ and cell inhibitor of apoptosis protein (cIAP).^62^

PROTACs afford multiple advantages compared with traditional small molecule inhibitors.^63^ First, PROTACs greatly expand the range of druggable proteins. More than 4000 disease-associated proteins have been identified. Among them, only ~400 proteins have been successfully exploited in current therapies. Many of them could not be targeted by traditional inhibitors due to their structural complexity, off-target effects and so on. Second, traditional inhibitors only block part of the protein’s function, while PROTACs degrade the protein, thus eliminating all its functions. Third, traditional kinase inhibitors often lead to drug resistance via mutations or overexpression of drug targets, but PROTACs could minimize drug resistance from long-term selection pressure by degrading target proteins. Last, PROTACs are active in a substoichiometric and catalytic manner, which allows them to function at low concentrations, thereby reducing possible toxic side effects.

### Molecular glue

Molecular glue facilitates the dimerization or colocalization of two proteins via forming a ternary complex.^64,65^ They can regulate a variety of biological processes, such as transcription, chromatin regulation, protein folding, localization, and degradation. The first examples of molecular glue are cyclosporin A (CsA) and FK506, which are used as immunosuppressants.^66^ Mechanistic studies reveal that CsA and FK506 induce the formation of cyclophilin-CsA-Calcineurin and FKBP12-FK506-Calcineurin complexes, respectively, giving rise to the term “molecular glues”.^66^ Subsequently, another immunosuppressive agent rapamycin was also discovered as a molecular glue by stabilizing the FKBP12-rapamycin-FRB (mTOR) ternary complex.^67^ In addition to immunosuppression, rapamycin and its analogs also exhibit antifungal, antitumor, and antiaging activities.

Molecular glue degraders induce the interaction between a ubiquitin ligase and a POI, leading to POI ubiquitination and subsequent degradation^68^ (Fig. 4b). Although both molecular glues and PROTACs harness the UPS for protein degradation, they have several distinctions (Fig. 4). First, PROTACs are heterobifunctional degraders that simultaneously interact with the E3 ligase and the POI; in contrast, molecular glue degraders could interact with only the ligase (more frequently) or the POI, and induce/stabilize their interactions. Second, molecular glues do not have a linker, making them smaller molecular weight, increased oral bioavailability, and improved cellular permeability, relative to PROTACs. Last, molecular glues are more difficult to design, although rational design strategies are emerging.

Examples of molecular glue degraders include thalidomide, lenalidomide, and pomalidomide.^69^ Interestingly, they have been approved by the FDA for the treatment of various types of tumors long before their functional mechanisms were elucidated. Years later, it was discovered that this class of compounds exert antitumor activities by acting as molecular glues^70^ (Fig. 3). They induce the interactions between E3 ligase, cereblon, and its transcription factor substrates IKZF1/3, leading to the degradation of IKZF1/3.^69^ With more drug-like properties, it is conceivable that molecular glues will receive more attentions from both academia and pharmaceutical industry.

### Double-mechanism degrader

**Fig. 5:**
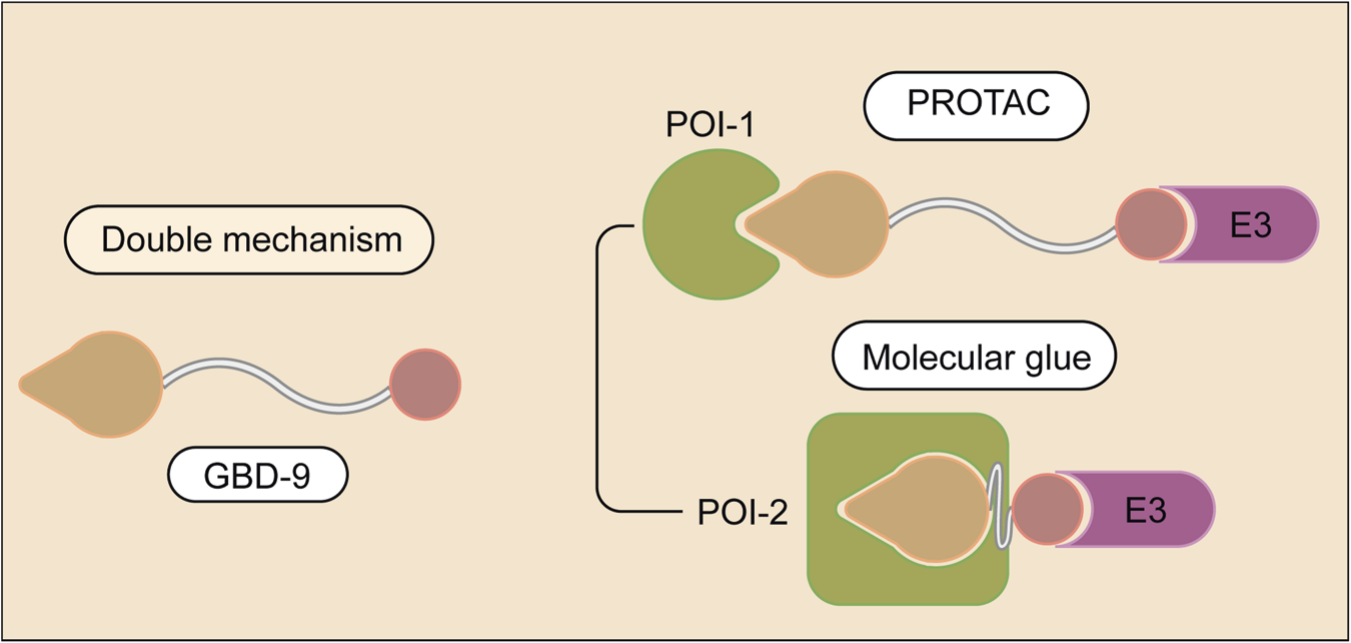
Schematic representation of a double-mechanism degrader. The molecule binds to one E3 ubiquitin ligase, and can degrade two different POIs via distinct mechanisms

Treatment of complicated diseases, such as cancer, often require more than one targets. Yang et al reported that a small molecule, GBD-9, that can target both bruton tyrosine kinase (BTK) and G1 to S phase transition 1 (GSPT1)^71^ (Fig. 5). BTK, a tyrosine kinase and a key regulator of the BCR (B-cell receptor) pathway, is up-regulated in a variety of lymphoma cells.^72^ GSPT1, a translation termination factor, is involved in regulation of mammalian cell growth.^73^ Interestingly, GBD-9 retains the characteristics of both a PROTAC and a molecular glue.^71^ The designer balanced the activities of PROTACs and molecular glues by modulating the length of the linker of BTK PROTACs. It appears that GBD-9 acts as a PROTAC to promote the degradation of RTK and, at the same time, as a molecular glue to promote the degradation of GSPT1^71^ (Fig. 5). As a consequence, GBD-9 displays stronger anti-proliferative effect in multiple cancer cell lines than ibrutinib, a small molecule BTK inhibitor. Future work will be needed to further illustrate the functional mechanism of GBD-9. As both PROTACs and molecular glues have strength and limitation, it will be excited to see more examples that can harness the strength of both strategies.

## Targeted protein degradation via lysosome

Lysosomes mediate the intracellular degradation of proteins and organelles in three different ways: endocytosis, phagocytosis, or autophagy^74^ (Fig. 2). Cells bring extracellular material or membrane proteins in via endocytosis.^75^ In phagocytosis, cells bind and engulf viruses, bacteria, or other large particles.^25^ Autophagy is a highly conserved cellular process in which misfolded or aggregated proteins, damaged organelles, and intracellular pathogens, are removed.^26,27^ There are three forms of autophagic pathways: macroautophagy, microautophagy, and chaperone-mediated autophagy (CMA).^26^ During macroautophagy, dysfunctional proteins or organelles are recognized by autophagy receptors and selectively enclosed in autophagosomes.^76^ Autophagosomes are then fuse with lysosomes and their contents are degraded. In microautophagy, lysosomes directly engulf autophagic cargo and lead to its degradation.^77^ In CMA, proteins are selected by chaperones, targeted to lysosomes, and directly translocated across the lysosome membrane for degradation. CMA has two unique features. First, CMA degrades only certain proteins, but not organelles. Second, the formation of autophagosomes is unnecessary in CMA.^78^

**Fig. 6:**
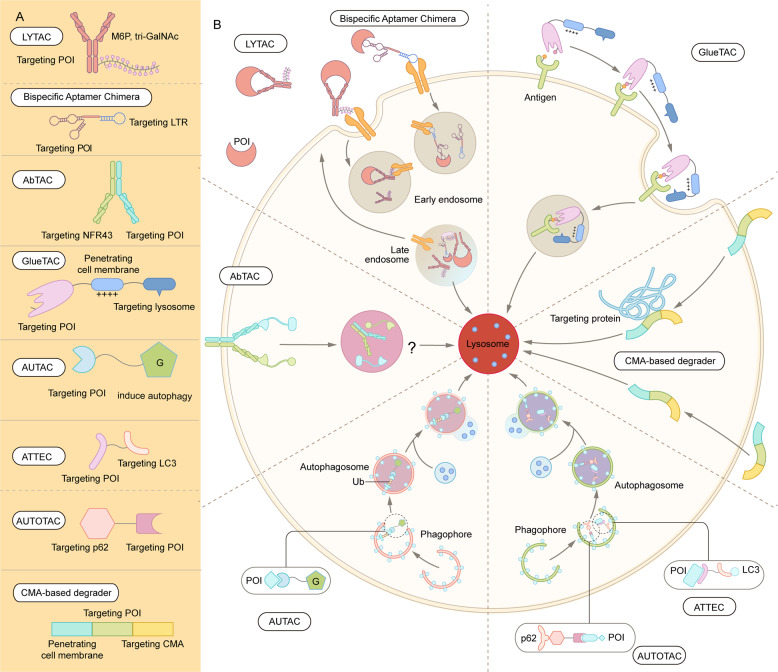
Summary of lysosome-dependent protein degradation strategies. **A** Schematic representation of LYTAC and other degradation technologies via the lysosomal pathway. **B** LYTAC molecules and Bispecific Aptamer Chimeras degrade membrane proteins and extracellular molecules by engaging a POI and a lysosome-targeting receptor (LTR). AbTAC utilizes a membrane E3 ligases, RNF43, for the degradation of membrane proteins, via a lysosome-dependent manner. GlueTAC utilizes a lysosome-sorting sequence (LSS) to promote the lysosomal degradation. AUTAC, ATTEC and AUTOTAC promote the formation of POI-specific autophagosomes, and subsequently the degradation of the POI via lysosomes. CMA-based degrader harness chaperone-mediated autophagy, rather than macroautophagy, for protein degradation

With the intensive research in the endosome-lysosome and autophagosome-lysosome degradation pathways, TPD strategies via the lysosomal pathway, such as LYTAC, AbTAC, ATTEC, AUTAC, bispecific aptamer chimeras, and AUTOTAC have emerged in recent years^39,79,80^ (Fig. 6 and Table 1). In contrast with proteasome-based TPD, which can only degrade certain intracellular proteins, lysosome-based TPD have potential to remove proteins aggregates, damaged excess organelles, membrane, and extracellular proteins.

### LYTAC

**Fig. 7:**
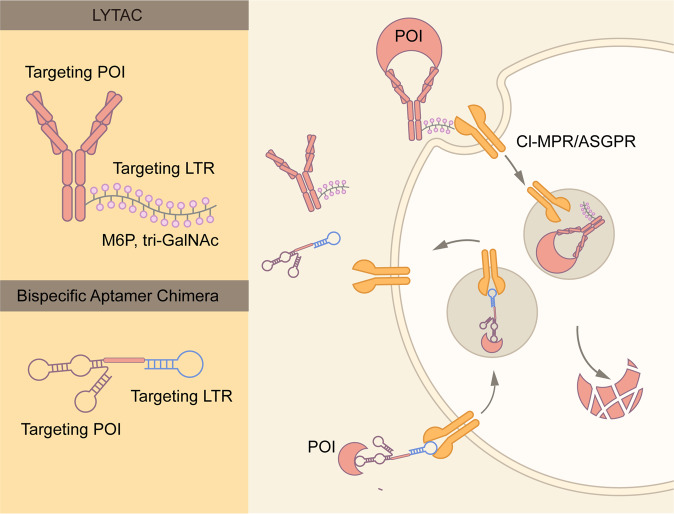
Schematic representation of LYTAC and Bispecific Aptamer Chimera. LYTAC is composed of a small molecule or an antibody conjugated to a ligand that binds to lysosome-targeting receptors (LTRs), such as CI-MPR and ASGPR. Whereas CI-MPR is ubiquitously expressed in all human tissues, ASGPR is only expressed in liver. Thus, ASGPR-base LYTAC could achieve liver specific protein degradation. CI-MPR or ASGPR is endocytosed along with LYTAC molecules and the POI. Whereas the POI is degraded by lysosomes, CI-MPR, or ASGPR is recycled to the plasma membrane for re-use. Bispecific Aptamer Chimera utilizes DNA aptamer to target the LTR and POI

LYTAC is a novel technique to induce the degradation of extracellular and membrane proteins via the endosome-lysosome pathway^81,82^ (Fig. 7). As extracellular and membrane proteins comprise 40% of the encoded proteins and are key contributors to neurodegenerative diseases, autoimmune diseases and cancer, LYTAC is a good complement to PROTACs. LYTAC molecules can simultaneously bind the extracellular domain of a membrane protein, or an extracellular protein, and a lysosome-targeting receptor (TLR) residing on the cell surface (Fig. 7). The formation of a ternary complex leads to protein internalization via clathrin-mediated endocytosis, and the POI is subsequently degraded.

The first reported LYTAC molecule is based on cation-independent mannose-6-phosphate receptor (CI-MPR), also known as IGF2R^81^ (Fig. 7). CI-MPRs facilitate intracellular trafficking of lysosomal enzymes, which are modified by N-glycans capped with mannose-6-phosphate (M6P).^83^ Low pH in late endosomes leads to the dissociation of lysosomal enzymes and CI-MPR. Whereas the former is targeted for lysosomal degradation, CI-MPR is transported to the Golgi apparatus and cell surface for recycling.^83^ This natural process is harnessed to generate the first LYTAC molecules, which consist of a small molecule or antibody fused with synthesized a CI-MPR-targeting ligand, poly-M6Pn.^81^ This LYTAC strategy has shown promises in degradation multiple therapeutically relevant proteins. For example, a LYTAC molecule derived by covalently conjugating poly-M6Pn to the EGFR antibody, cetuximab, was shown to specifically degrade EGFR in a variety of cell lines.^81^ In addition, conjugation of poly-M6Pn with anti-PD-L1 antibody led to a significant decrease of PD-L1 at the cell surface.^81^

Whereas the expression of CI-MPR is ubiquitous, the expression of certain LTRs is tissue specific. Molecules that target tissue-specific LTRs could induce the degradation of target proteins in specific tissues. Asialoglycoprotein receptor (ASGPR) is a liver-specific LTR.^84,85^ The ASGPR-based LYTAC molecule is made by the fusion of antibodies with N-acetylgalactosamine (GalNAc), that target ASGPR^82^ (Fig. 7). Co-culture experiments demonstrate that this LYTAC technology specifically targets cells that express ASGPR.^82^ With initial success of CI-MPR- and ASGPR-based LYTAC, the search for other LTRs is warranted.

### Bispecific aptamer chimera

Similar to LYTAC, Bispecific Aptamer Chimera also mediates the degradation of POI via the endosome–lysosome pathway^79^ (Fig. 7). In contrast with LYTAC, Bispecific Aptamer Chimera utilizes DNA aptamer targeting CI-MPR and the transmembrane POI (Fig. 7). The Han team designed the first Bispecific Aptamer Chimera molecule named A1-L-A2, in which A1 and A2 specifically bind to CI-MPR and a POI, and L stands for a linker DNA.^79^ The aptamer chimeras could shuttle membrane proteins, such as receptor tyrosine kinase MET and PTK-7, to lysosomes for degradation.^79^ At the same time, the aptamer chimeras had no significant effect on the levels of non-targeting proteins. Overall, this method provides a powerful, efficient, and versatile platform to induce the degradation of membrane proteins. Nucleic acid aptamers have many advantages relative to antibodies, including simple preparation, precise synthesis, and stability.

### AbTAC

**Fig. 8:**
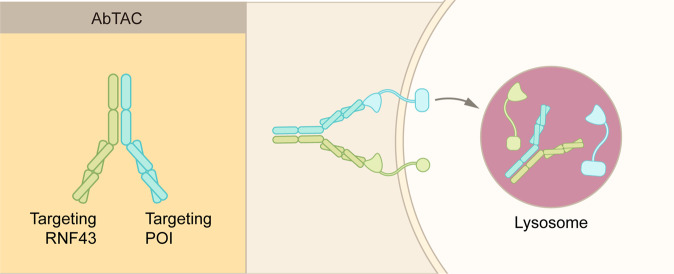
Schematic representation of AbTAC. AbTAC utilizes recombinant bispecific antibody to recruit a membrane protein and a membrane-bound E3 ligase, RNF43. The POI is likely degraded via the lysosomes, but not by the proteasomes. However, the exact mechanisms remain to be established

Antibody-based PROTAC (AbTAC) is another emerging TPD technology that induces the degradation of extracellular and membrane proteins^86^ (Fig. 8). Compared to conventional PROTAC, AbTAC can target membrane proteins, thus, greatly extending the potential substrates of current TPD strategies. Although bearing the name of PROTAC, AbTAC is more closely related with LYTAC. AbTAC utilizes bispecific antibodies, with one arm targeting a cell-surface POI, and the other arm targeting a transmembrane E3 ligase, such as RNF43^87^ (Fig. 8). The addition of AbTAC molecule induces the complex internalization and subsequent lysosomal degradation of the POI.

Similar to LYTAC, AbTAC also mediate the TPD of cell-surface POI by harnessing the endosome–lysosome pathway (Figs. 6–8). However, the mechanism of action of AbTAC is less clear than that of LYTAC. Particularly, it is unknown whether the intracellular region of a POI is ubiquitinated prior to endocytosis; if it does, how the ubiquitination contribute to the complex internalization. Furthermore, it remains unknown whether RNF43 could be recycled and re-used like LYTAC receptors, CI-MPR and ASGPR. In addition to RNF43, additional membrane receptors need to be identified for the development of AbTAC technology.

### GlueTAC

**Fig. 9:**
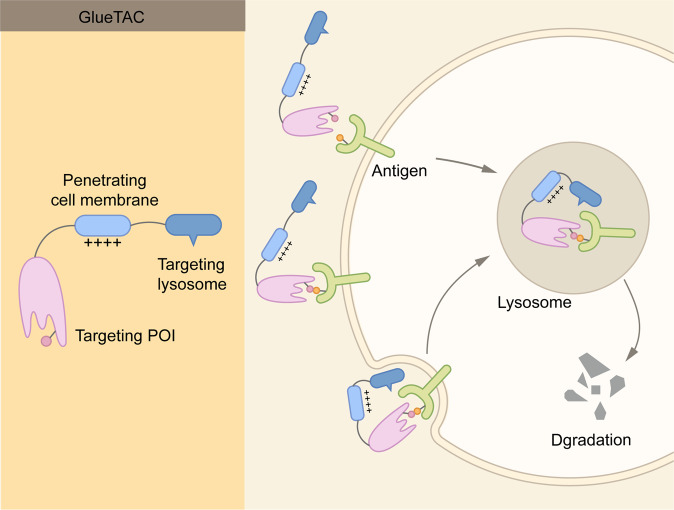
Schematic representation of GlueTAC. GlueTAC consists of a covalently-modified nanobody, a Cell-penetrating peptide (CPP), and a lysosome-sorting sequence. The nanobody is responsible for targeting POI, and the CPP induces endocytosis of the GlueTAC-POI complex and subsequent lysosomal degradation

Recently, another lysosome-based strategy, termed GlueTAC, has been developed to degrade cell-surface proteins^88^ (Fig. 9). GlueTAC utilizes three major technologies to facilitate the degradation. First, nanobodies are used to replace conventional antibodies to facilitate cell penetration. Second, covalent interaction is introduced between nanobodies and antigen to overcome relatively low binding affinity and to minimize off-target effects. Third, a cell-penetrating peptide and lysosome-sorting sequence (CPP-LSS) is conjugate to the nanobodies to promote the internalization and lysosomal degradation^89,90^ (Fig. 9). To demonstrate the effectiveness of GlueTAC, the authors developed a GlueTAC molecule targeting PD-L1.^88^ This GlueTAC molecule is more effective in reducing the level of PD-L1 in cells and inhibiting tumor growth in immunodeficient mice, in comparison with FDA-approved antibody against PD-L1, Atezolizumab.^88^

Whereas GlueTAC represent another exciting approach in degrade cell-surface proteins, several issues need to be considered. First, the safety. GlueTAC introduces unnatural amino acids in nanobodies and creates covalent bonds between nanobodies and antigens. Thus, the safety of GlueTAC molecules need to be carefully assessed. Second, the nanobodies do not have heavy chains and cannot bind to FcRn.^91,92^ The half-life of GlueTAC also needs to be determined.

### AUTAC

**Fig. 10:**
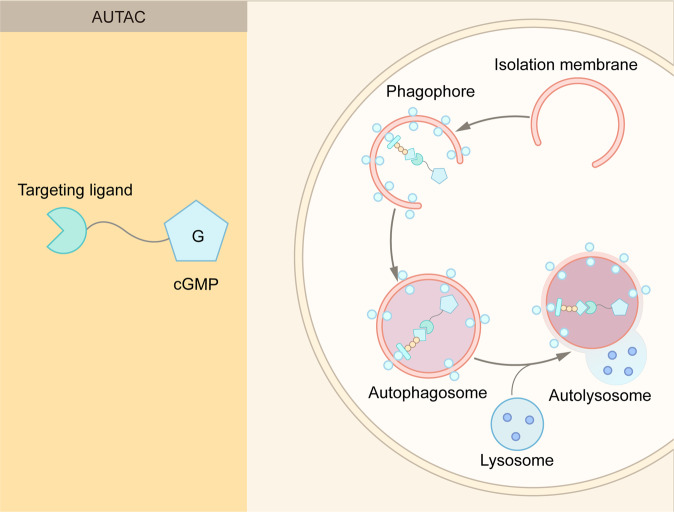
Schematic representation of AUTAC. AUTAC molecules consist of a POI-targeting warhead, a linker, and a cGMP-based degradation tag. The degradation tag recruits autophagosomes to degrade cytoplasmic proteins and cellular organelles

In addition to the endosome–lysosome pathway, the autophagy–lysosome pathway provides another avenue for TPD^93,94^ (Fig. 6 and Table 1). Nucleotide 8-nitrocyclic guanosine monophosphate (8-nitro-cGMP) is an important signaling molecule in cells to mediate the recruitment of autophagosomes.^95^ This property of 8-nitro-cGMP was used for the development of autophagy-targeting chimera (AUTAC) (Fig. 10). An AUTAC molecule consists three parts: a cGMP-based degradation tag, a linker, and a small molecule ligand for a POI or an organelle.^93^ An AUTAC molecule triggers K63-linked polyubiquitin, and subsequent lysosome-mediated degradation (Fig. 10). In contrast, a PROTAC molecule induces K48-linked polyubiquitin and proteasome-mediated degradation.

In addition to cytoplasmic proteins, cellular organelles such as mitochondria could be degraded via AUTAC.^93^ Mitochondrial dysfunction is associated with many aging-related diseases, and the removal of dysfunctional or damaged mitochondria may ameliorate these diseases. Takahashi et al developed a molecule known as AUTAC4, which promotes mitophagy of fragmented mitochondria.^93^ AUTAC4 utilizes a 2-phenylindole derivative, which is a ligand for a transporter on the outer mitochondrial membrane, as a mitochondria binder. The treatment of AUTAC4 was shown to restore mitochondrial membrane potential and ATP production.^93^ These results indicate broad applications of AUTAC, and it is expected to see more interesting applications of AUTAC, such as the degradation of protein aggregates.

### ATTEC

**Fig. 11:**
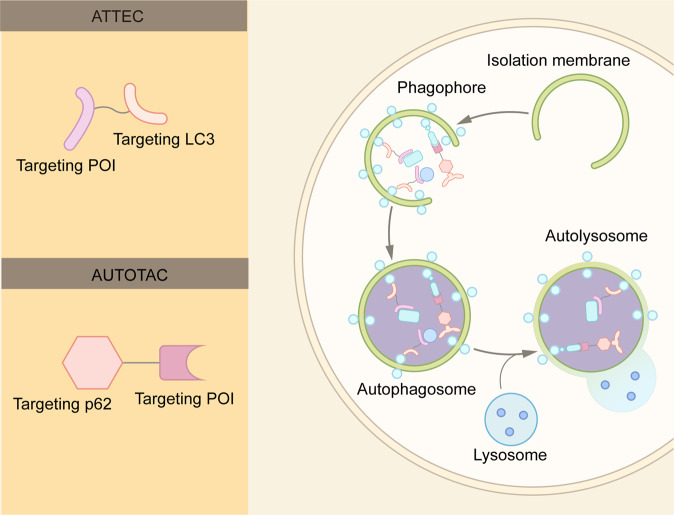
Schematic representation of ATTEC and AUTOTAC. An ATTEC molecule simultaneously binds LC3 and a POI, while an AUTOTAC molecule binds p62 and a POI. The binding induces the formation of autophagosomes, and subsequent fusion between autophagosomes and lysosomes lead to the POI degradation

Similar to the autophagy-based AUTAC, autophagosome tethering compound (ATTEC) functions by tethering the POI to the autophagosome^96,97^ (Figs. 6 and 11). Whereas AUTAC recruits autophagosomes for degradation, ATTEC binds to LC3, one of the key proteins of autophagosome.^98,99^ Lu and coworkers discovered a set of small molecules that are capable binding of LC3 protein and pathogenic mutant huntingtin proteins.^96^ Remarkably, these molecules can distinguish wild-type and mutant huntingtin proteins,^96^ which are identical except for the length of the polyglutamine (polyQ) stretch. Mutant huntingtin protein has at least 36 glutamines. The longer the polyQ stretch, the earlier symptoms typically appear.^100^ The researchers proposed that these molecules recognize the conformation of the expanded polyQ stretch in the mutant protein and distinguish them from the wild-type protein.^97^ By specifically recognizing the mutant huntingtin protein, ATTEC provides new possibility for the treatment of Huntington disease. Furthermore, it will be interesting to determine whether these ATTEC molecules can be used for other polyQ diseases, such as dentatorubral pallidoluysian atrophy and Machado-Joseph disease.^101^

Recently, Lu and colleagues further extend the application of ATTEC by developing small molecules targeting Lipid droplets (LD-ATTEC), the fat-storage organelles in cells.^97^ These compounds bind LC3 protein as well as Lipid droplets, and can reduce the number of Lipid droplets at micromolar concentrations. Furthermore, they can rescue LD-related phenotypes in two independent mouse models.^97^ Collectively, these studies demonstrate that ATTEC could harness the autophagy-lysosome pathway for the degradation of proteins and non-protein materials.

### AUTOTAC

The autophagy cargo receptor p62/SQSTM1 functions to bridge polyubiquitinated cargo and autophagosomes.^102^ Polyubiquitinated cargos bind to the UBA domain of p62, leading to a conformational change in p62. Such a conformation change exposes the LIR motif of p62, and prmotes its interaction with LC3 on the autophagic membrane. Ji et al. designed the AUTOphagy-TArgeting Chimera (AUTOTAC) platform that bypasses the requirement of ubiquitin^80^ (Fig. 11). AUTOTAC molecules consist of a module that interacts with the ZZ domain of p62, and a POI-targeting module.^80^ The addition of AUTOTAC molecules bridges the POI and p62, independent of ubiquitin on the POI. AUTOTAC promotes the oligomerization and activation of p62, leading to the degradation of the POI by the autophagy–lysosome pathway (Fig. 11).

AUTOTAC can mediate the targeted degradation of not only monomeric proteins, but also aggregation prone proteins. Using murine models expressing human pathological tau mutants, Ji et al demonstrated the AUTOTAC could effectively remove misfolded tau.^80^ In contrast, the proteasome-based technologies, such as PROTAC and molecular glue, are usually ineffective in dealing with the misfolded proteins. In addition to Tau, AUTOTACs could also efficiently remove multiple oncoproteins, such as degrading androgen receptor (AR).^80^

### CMA-based degrader

**Fig. 12:**
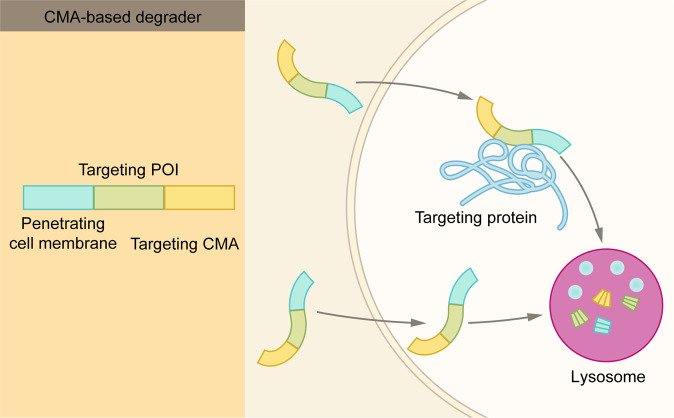
Schematic representation of CMA-based degrader. CMA-based degrader consists of three modules: a CMA-targeting module, a cell-penetrating peptide, and a POI-targeting module. After the CMA-based degrader entering the cell, it binds the POI and induces chaperone-mediated autophagy

In chaperone-mediated autophagy, heat shock protein 70 (HSC70) recognizes soluble protein substrates with KFERQ sequence.^103^ The HSC70-substrate complex then binds to lysosomal associated membrane protein 2A (LAMP2) on the lysosomal membrane, and the substrate is then translocated to lysosome lumen for degradation.^104^ Thus, a chimera peptide containing the KFERQ sequence and a targeting protein-binding sequence could be exploited to degrade pathogenic or misfolded proteins. CMA-based degraders include three functional domains: a cell membrane penetration sequence, a POI-binding sequence, and a CMA-targeting motif^105^ (Fig. 12). Upon the addition to the cells, a CMA-based degrader first enters the cell, then binds to the target protein via the POI-binding sequence, and finally transports to the lysosomes for degradation.^105^ Indeed, this strategy has been shown to reduce the levels of mutant huntingtin protein, PSD-95, death-associated protein kinase 1 (DAPK1), as well as α-synuclein.^105,106^

To become an effective therapeutic strategy, CMA-based degraders need to overcome at least two major hurdles. First, the stability of the degrader. Second, the delivery efficiency. Overall, whereas the CMA-based degraders represent a new approach in TPD, they face great challenges that are not seen by other TPD technologies, such as PROTAC and LYTAC.

## Application of targeted protein degradation in disease treatment

The last few years have seen explosive growth in the field of TPD.^107^ Currently, about ten TPD molecules are in the cancer clinical trials.^108^ In addition to cancer, many TPD molecules show great promising for the treatment of neurodegenerative diseases, inflammatory diseases, or viral infection.

### Cancer

Multiple TPD molecules (mostly based-on the PROTAC technology) have shown potential therapeutic effects in cancer clinical trials and preclinical studies.^109^ Due to the space limitation, we can only highlight a few examples. The estrogen receptor (ER) is a master regulator of gene expression, and is critical for the pathogenesis of breast cancer.^110–112^ ARV-471 is a PROTAC molecule developed by Arvinas and specially target ER.^113^ In preclinical experiments, ARV-471 leads to efficient ER degradation and significantly reduces tumor burden in xenograft models.^113^ Now in phase II clinical trial, ARV-471 can be given either as a single agent or in combination with a CDK4/6 inhibitor. In clinical experiments, ARV-471 shows good oral bioavailability and favorable tolerability.

In addition to ARV-471, ARV-110 is another PROTAC small molecule entering phase II clinical trial.^108^ ARV-110 selectively targets androgen receptor (AR) and leads to its degradation. ARV-110 is developed as a potential treatment for prostate cancer, the second most common malignancy in men after lung cancer.^108^ Most notably, ARV-110 shows great efficacy in patients whose tumors harbored the AR T878X/H875Y (T878X = T878A or T878S) mutations and are resistant to currently available AR-targeted therapies. These results further emphasize that TPD has become a promising approach in pharmaceutical industry by targeting these “undruggable” targets.

Signal transducer and activator of transcription 3 (STAT3) is constitutively activated in a variety of human cancers.^114–118^ Small-molecule inhibitors targeting the STAT3 SH2 domain have selectivity problems and show very limited clinical activity. Recently, a potent and specific PROTAC degrader of STAT3, SD-36, was developed.^119,120^ SD-36 is made of analog of CRBN ligand lenalidomide, a linker and the STAT3 inhibitor SI-109.^121^ SD-36 efficiently and rapidly degrades STAT3 in leukemia and lymphoma cell lines. Interestingly, SD-36 is highly selective for STAT3 among all the STAT proteins. Furthermore, SD-36 also achieves a robust and long-lasting STAT3 degradation in multiple xenograft mouse models.^119^

BCL-XL, a member of the BCL-2 family, protects cancer cells from programmed cell death.^122,123^ Small molecule inhibitors targeting the BCL-2 class proteins, such as ABT263 (BCL-2 and BCL-XLdual inhibitor) and ABT199 (BCL-2 selective inhibitor), have been developed.^124^ However, these molecules often have significant side effects, thus limiting their utility. Recently, a potent BCL-XL PROTAC molecule, DT2216, was developed by conjugation ABT263 with a VHL ligand.^125,126^ DT2216 leads to robust BCL-XL degradation in tumor cells. It also displays reduced side effects relative to ABT263, likely due to the low expression of VHL in platelets. Interestingly, DT2216, although binding to BCL-XL and BCL-2 with similar affinity, does not induce the degradation of BCL-2.

### Neurodegenerative diseases

Neurodegenerative diseases (NDs) are a group of disorders characterized by progressive motor or cognitive impairment.^127^ NDs, including Alzheimer’s disease (AD), Parkinson’s disease (PD), and Huntington disease (HD), are closely associated with insoluble aggregates formed by protein misfolding.^128^ Misfolded proteins often display unusual protein-protein interactions (PPIs) that are unrelated with their normal functions.^129,130^ Traditional drug discovery is often based on modulating the functions of target proteins. As a result, novel drug discovery modes, such as TPD, are urgently needed in order to develop therapeutic approaches for NDs.^131,132^

In 2016, Chen and Li groups reported a PROTAC molecule targeting tau protein, the first attempt to apply the PROTAC technology for the treatment of NDs.^133^ The PROTAC molecule they designed is a chimera construct made of a tau-binding peptide, a linker, a VHL-binding peptide, and a cell-penetrating peptide.^133^ This molecule leads to a significant degradation of tau and reduced neurotoxicity of Aβ. Another polypeptide PROTAC for AD was developed by Jiang et al., who used a CRLKeap1-binding sequence.^134^ This molecule also successfully achieved tau protein degradation.

### Inflammatory diseases

In addition to cancer and neurodegenerative diseases, the reach of TPD has extended to inflammatory diseases and immuno-oncology. IRAK-4 (interleukin-1 receptor-associated kinase 4) is a member of the IRAK kinase family and involved in Toll-like receptor (TLR) and IL-1R signaling pathways.^135^ Upon TLR activation, IRAK-4 is recruited to form the Myddosome complex, which subsequently leading to the phosphorylation of other members of the IRAK family, such as IRAK1 and IRAK2.^136^ In addition to its enzymatic activity, the scaffolding role of IRAK-4 in TLR signals is also well established.^137^ In comparison with conventional inhibitors, IRAK-4 degraders provide great advantages by eliminating both enzymatic and non-enzymatic functions of IRAK-4. Indeed, multiple IRAK-4-targeting PROTAC molecules have been developed, with one entering phase I clinical trials to treat autoimmune diseases.^138–140^

BTK is an established target in both inflammation and cancer.^141^ Although BTK inhibitors have been proved and used in the clinic to treat different hematological cancers, such as leukemia and lymphoma, the appearance of BTK mutations renders these drugs less effective. These challenges could be uniquely addressed by BTK degraders as these molecules may degrade both wide-type and mutant BTK proteins.^142,143^ Two BTK PROTACs are currently in a phase I trial for the treatment of B cell malignancies and autoimmune diseases.^107^

### Viral infection

Viral infection poses a great challenge in global health. SARS-CoV-2 is one of the worst examples, which have infected over 400 million individual and killed 5.7 million worldwide.^144,145^ TPD could represent a novel antiviral therapeutic approach. One of the first successful examples is used for the degradation of hepatitis C virus (HCV) NS3/4A protease. de Wispelaere et al.^146^ showed that telaprevir (the HCV protease inhibitor)-based PROTACs could inhibit HCV in a cellular infection model. Currently, there are great interests in the development of PROTACs that target SARS-CoV-2 across academia and industry.^107^ In addition to PROTAC, technologies targeting the autophagy–lysosome pathway, such as AUTAC and ATTEC, could be also used to eliminate key viral proteins.^132^

## Summary and outlook

The past two decades have seen the birth and boom of the TPD technologies. PROTAC and molecular glue are the most advanced TPD technology. Both are based on the ubiquitin-proteasome system and useful for the degradation of intracellular proteins. In the past 5 years, technologies harnessing the second degradation pathway in cells have emerged and quickly developed. These technologies can be further divided into two groups based on their degradation mechanisms. LYTAC, Bispecific Aptamer Chimeras, AbTAC, and GlueTAC, degrade extracellular and membrane proteins by harnessing the endosome-lysosome pathway. In addition, technologies targeting the autophagy-lysosome pathway, such as AUTAC, ATTEC, AUTOTAC, and CMA chimeras, can degrade misfolded proteins, protein aggregation, or damaged organelles.

Multiple PROTAC molecules, including cancer drug candidates ARV-110 and ARV-471, have shown great promising in clinical trials. Nevertheless, the PROTAC technology, as a whole, still faces many challenges. First of all, pharmaceutical properties. PROTAC molecules often face the challenges of cell permeability and oral bioavailability due to their large size. Molecular glues are smaller and have some advantages over PROTAC molecules; however, they are more difficult to rationally design. Second, the repertory of E3 ubiquitin ligase. Human genome encodes more than 600 E3 ubiquitin ligases, and only a few of them (VHL, CRBN, IAPs, and MDM2) have been utilized to degrade target proteins. Third, toxicity. PROTAC could result in more toxicity than small molecular inhibitors because they degrade entire targeted proteins, rather than solely inhibit them.

Relative to PROTAC and molecular glue, the development of lysosome-based TPD technologies is still in the infancy stage. We still have much to learn about the specific mechanism of each technology. As an important intracellular organelle, lysosomes regulate many important cellular and physiological functions in addition to protein degradation, such as the metabolism and homeostasis. It is unclear whether “hijacking” lysosomal degradation pathway will affect the body as a whole. Expanding the repertory of lysosome-targeting receptors, which currently include CI-MPR and ASGPR only, is much needed for LYTAC and similar technologies. Further characterization of AUTAC, ATTEC, and AUTOTAC molecules, including systematic study their structure−activity relationship and understanding of their modes of action, is necessary. These efforts will help to develop the autophagy-based technologies as a general method for protein degradation, analogous to PROTAC. Current CMA-based degraders are mostly limited by cell membrane permeability and stability, and their small-molecule forms may overcome these obstacles. Lysosome-based technologies have greatly broadened the spectrum of targets by PROTAC and molecular glue, and a surge of interest in this field is definitely expected. Despite these challenges, TPD technologies, undoubtedly, will not only provide powerful tools for biomedical research, but hold great promise for future drug development.
